# Impact of Lipoma Size and Depth on Local Anesthetic Volume: Exploring the Efficacy of Diluted Lidocaine for Excision

**DOI:** 10.7759/cureus.94586

**Published:** 2025-10-14

**Authors:** Yuto Yamamura, Kazuyasu Fujii, Chisa Nakashima, Kazutoshi Nishimura, Atsushi Otsuka

**Affiliations:** 1 Dermatology, Kindai University, Osaka, JPN; 2 Dermatology, Kindai University Hospital, Osaka, JPN

**Keywords:** lidocaine dilution, lipoma excision, outpatient excision, tumescent anaesthesia, under local anesthesia

## Abstract

Local anesthesia is commonly used in the surgical excision of lipomas. However, concerns regarding anesthetic toxicity and insufficient analgesia may arise in larger or deeper tumors. Tumescent anesthesia using highly diluted lidocaine has been proposed to mitigate these risks, though its clinical utility remains insufficiently studied. In this retrospective observational study, we evaluated the efficacy and safety of 10-fold diluted 1% lidocaine in the excision of 53 lipomas from 38 patients. We also investigated how tumor characteristics, including size and depth, influenced anesthetic volume and the choice of anesthesia (local vs. general). Local anesthesia was used in 86.8% of cases, whereas general anesthesia was typically reserved for deep-seated lipomas. Among cases performed under local anesthesia, total anesthetic volume was positively correlated with lipoma size (ρ = 0.666, p < 0.001), while the volume of undiluted 1% lidocaine showed only a weak correlation (ρ = 0.280, p = 0.084). Ten-fold diluted lidocaine was effective for providing adequate analgesia, even for large tumors, and its use reduced the need for general anesthesia. Our findings suggest that the use of highly diluted lidocaine is a safe and practical method in the outpatient excision of lipomas, especially when deeper lesions are avoided. This approach may help optimize anesthetic strategies by minimizing lidocaine dosage while maintaining analgesic efficacy. While lipoma depth remains a critical factor in anesthesia selection, the broader application of diluted local anesthetics could expand the role of local anesthesia in soft tissue surgery.

## Introduction

Lipomas are among the most common soft tissue tumors, typically presenting as painless, slow-growing masses [1,2]. Surgical excision is often performed for cosmetic reasons, relief of discomfort, or improvement of functional limitations.

Lidocaine, one of the most widely used local anesthetics, may induce local anesthetic systemic toxicity (LAST), including central nervous system manifestations and cardiotoxicity, if administered in doses exceeding the maximum safe limit [3]. Consequently, concerns regarding excessive anesthetic dosage in the management of large lipomas have led to the use of general anesthesia in some cases [4].

In addition, lipomas arising in certain anatomical locations, such as the forehead or flank, may extend into deeper tissues. These so-called deep-seated lipomas pose greater surgical challenges, and general anesthesia may be considered when local anesthesia proves inadequate [5].

On the other hand, previous reports have demonstrated that by employing tumescent anesthesia--originally developed for liposuction and characterized by highly diluted lidocaine solutions--the risk of systemic toxicity can be minimized, allowing even large lipomas to be excised under local anesthesia [4,6]. Nevertheless, the extent to which lipoma size and depth influence anesthetic choice and the required anesthetic volume remains unclear, and the analgesic efficacy of diluted lidocaine has not been thoroughly evaluated.

The present study aimed to evaluate the relationship between lipoma size and the total anesthetic volume required for excision as its primary objective. The secondary objective was to assess the analgesic efficacy of a 10-fold diluted 1% lidocaine solution and its potential to reduce the total lidocaine dosage, thereby providing new insights into anesthetic strategies for lipoma excision.

## Materials and methods

Study population and exclusion criteria

A total of 38 patients who underwent lipoma excision at our institution between April 2022 and December 2024 were included. Cases involving multiple lipomas in which the exact volume of local anesthetic used for each lesion was unclear (n = 1) and cases performed under general anesthesia (n = 5) were excluded from the analysis of local anesthetic usage.

Data collection and analysis

Lipoma cases were identified from surgical records, and patient information and operative details were extracted from electronic medical records. Lipoma volume (V, cm³) was calculated by measuring three dimensions (length a, width b, thickness c) and applying the formula:

\[ V = \frac{\pi}{6} \times a \times b \times c \]

(where π was approximated as 3.14)

For local anesthesia, 1% lidocaine with 1:100,000 epinephrine was diluted in saline as needed. To adjust the pH, approximately 5% sodium bicarbonate (8.4%) was added.

Total local anesthetic volume was recorded by the assisting nurse for each procedure. All local anesthetic was injected around the lipoma (e.g., in the subcutaneous tissue), similarly to a typical field block, rather than directly into the lipoma.

Statistical analysis

Spearman’s rank correlation was used to assess the relationship between lipoma volume and anesthetic usage. Group comparisons were carried out with the Kruskal-Wallis test or Mann-Whitney U test, with a p-value < 0.05 considered significant. Analyses were performed using R version 4.4.2.

## Results

Patient and tumor characteristics

A total of 61 lipomas were excised from 38 patients, of whom 6 (15.8%) had multiple lipomas. The patient age ranged from 30 to 93 years (median, 57 years). Lipomas were most commonly located in the lower back or dorsal region (19 (31.1%)), followed by the head and neck (18 (29.5%)), lower extremities (9 (14.8%)), upper extremities (7 (11.5%)), thoracoabdominal region (5 (8.2%)), and thoracic/dorsal region (3 (4.9%)). Lipoma volumes ranged from 0.30 cm³ to 388.58 cm³ (median, 17.27 cm³). 6 (9.84%) lipomas extended into deeper structures (e.g., intermuscular space).

Anesthesia choice

**Figure 1:**
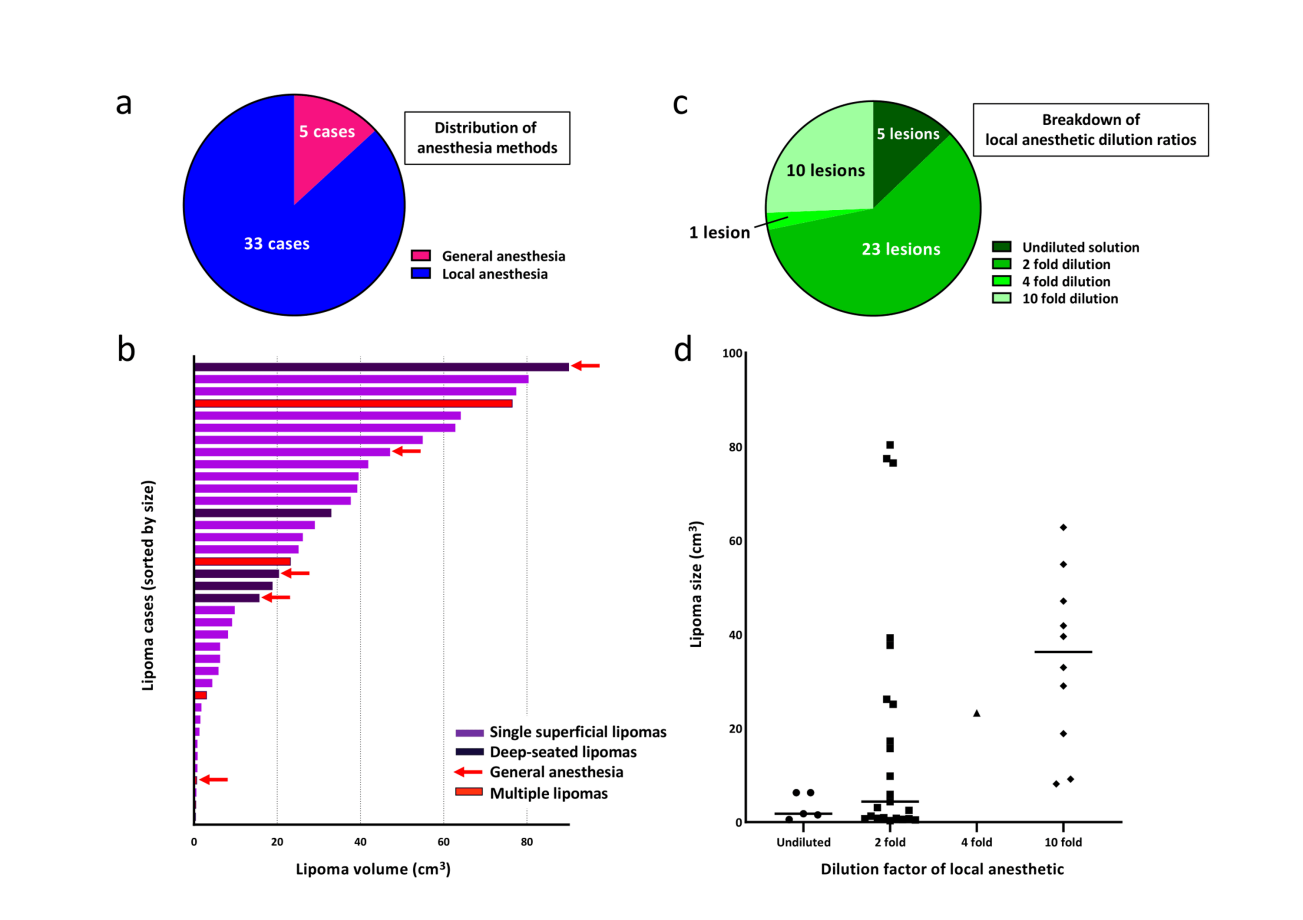
Anesthesia methods and local anesthetic dilution ratios (a) Lipomas from 38 cases arranged in descending order of size. Deep-seated lipomas are shown in black, and cases under general anesthesia are marked with red arrows; for multiple lipomas, only the largest is shown. (b) Distribution of anesthesia methods: local anesthesia in 86.8% (33/38) vs. general anesthesia in 13.2% (5/38). (c) Relationship between lipoma size and dilution ratio. Larger lipomas were more likely to receive higher dilution ratios (p = 0.0012). (d) Proportions of each dilution ratio used: undiluted (5/39), 2-fold (23/39), 4-fold (1/39), and 10-fold (10/39).

Local anesthesia was used in 33 (86.8%) cases, whereas 5 (13.2%) cases required general anesthesia (Figure 1a). Among those who underwent local anesthesia, the median volume of undiluted 1% lidocaine was 4.00 mL (IQR: 2.35-5.45 mL), while the median total anesthetic volume (including diluent) was 8.4 mL (IQR: 5.5-11.7 mL).

General anesthesia vs. local anesthesia

Among the five cases requiring general anesthesia, four involved deep-seated lipomas. There was no significant difference in lipoma size between the general anesthesia and local anesthesia groups (p = 0.489). However, deep-seated lipomas were more likely to be excised under general anesthesia (Figure 1b).

Lipoma volume and local anesthetic usage

Of the 33 cases performed under local anesthesia, 32 (97.0%) cases had detailed usage data, covering 39 lipomas. The dilution ratios were 5 (12.8%) lipomas for undiluted, 23 (59.0%) for 2-fold, 1 (2.6%) for 4-fold, and 10 (25.6%) for 10-fold (Figure 1c). A Kruskal-Wallis test (H = 12.53, p = 0.0057) indicated a significant difference in lipoma volume among the dilution groups, with post hoc comparisons showing significant differences between the 1-fold group (1 (100%)) and 10-fold group (10 (100%)) (p = 0.012), as well as between 2-fold group (2 (100%)) and 10-fold group (10 (100%)) (p = 0.048), while no significant differences were found among the other groups (Figure 1d).

Undiluted lidocaine usage per cm³ of tumor volume

**Figure 2:**
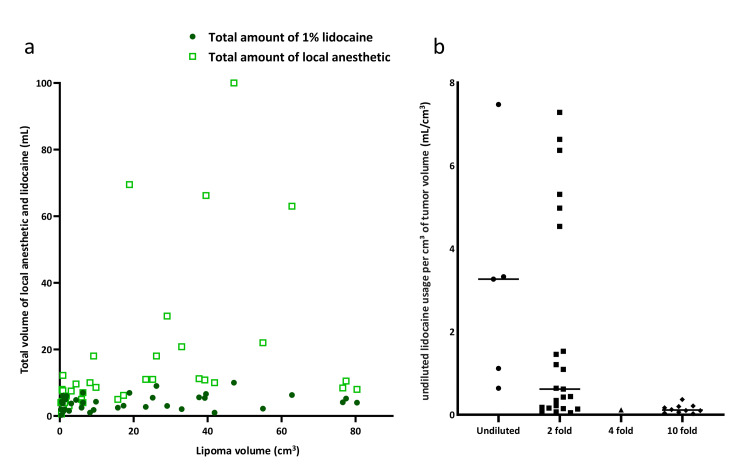
Lipoma size and local anesthetic usage (a) Correlation between lipoma size and both total anesthetic volume (p < 0.001) and 1% lidocaine volume (p = 0.084). Lipoma size was positively correlated with total volume but not with the absolute volume of lidocaine. (b) Undiluted lidocaine usage per cm³ of tumor volume by dilution ratio, demonstrating significantly greater efficiency in the 10-fold dilution group compared with undiluted or 2-fold dilution.

Spearman’s rank correlation indicated a moderate positive correlation between lipoma size and the total volume of diluted anesthetic solution administered (ρ = 0.666, p < 0.001), but no significant correlation between lipoma volume and the volume of undiluted 1% lidocaine used for preparation (ρ = 0.280, p = 0.084) (Figure 2a). Comparing the ratio of undiluted lidocaine volume to lipoma volume across dilution groups revealed significantly higher undiluted lidocaine usage per cm³ of tumor volume in the 10-fold dilution group (p < 0.05) (Figure 2b).

Additional local anesthesia

**Figure 3:**
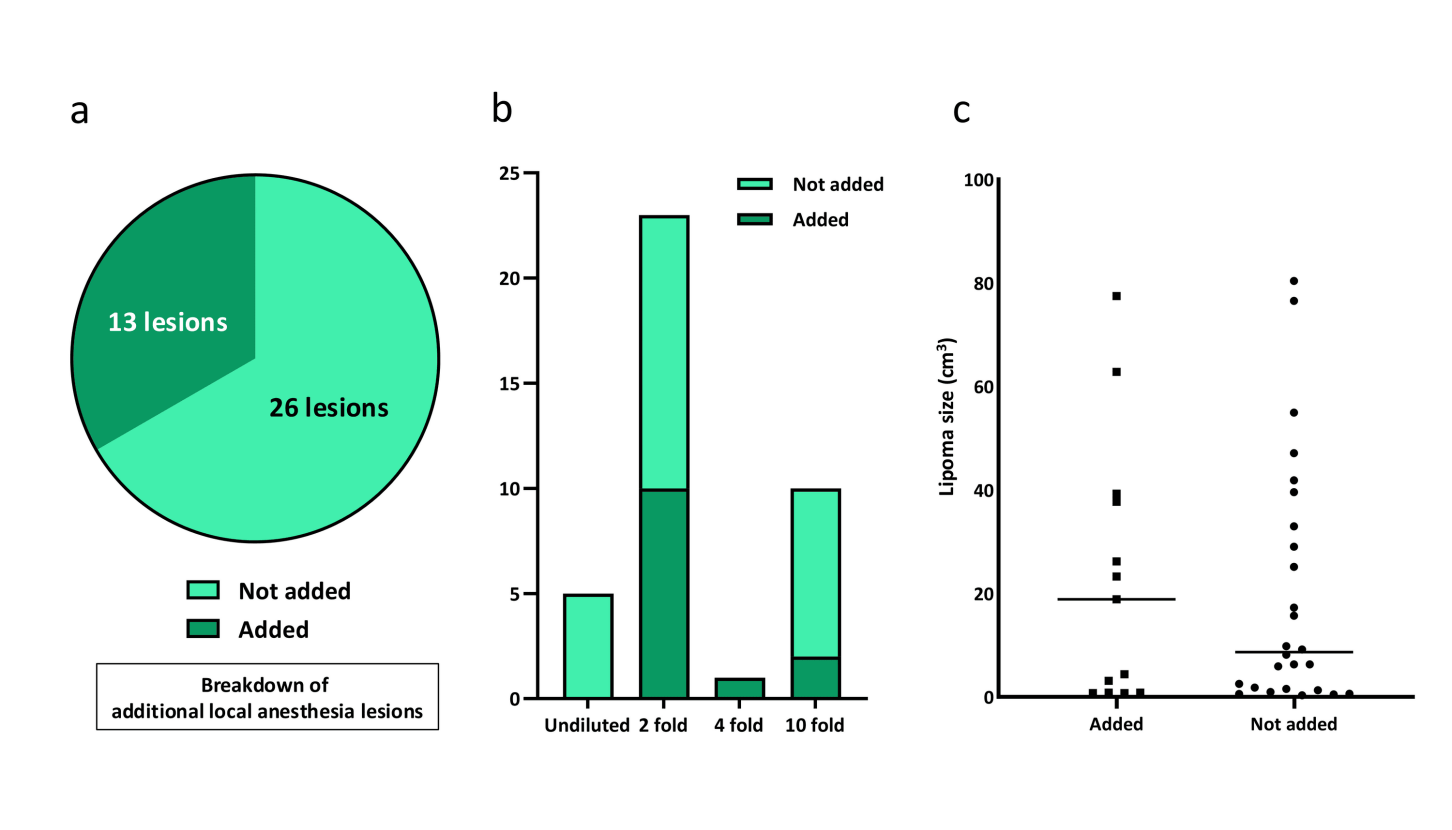
Additional local anesthesia requirements (a) Overall proportion of lipomas requiring additional local anesthesia (33.3%, 13/39). (b) Breakdown by dilution ratio: undiluted (0/5, 0%), 2-fold (10/22, 45.5%), 4-fold (1/1, 100%), 10-fold (2/10, 20%). (c) Lipoma size showed no significant difference (p = 0.772) between cases with and without additional local anesthesia.

Overall, 13 (33.3%) lipomas required additional local anesthesia due to insufficient pain control (Figure 3a). The proportion of cases needing additional anesthesia varied by dilution ratio: 0 (0%) in the undiluted group, 10 (45.5%) in the 2-fold group, 1 (100%) in the 4-fold group, and 2 (20%) in the 10-fold group (Figure 3b). Among the 2 deep-seated lipomas, 2 (100%) required additional anesthesia. Lipoma volume did not differ significantly between cases with or without additional anesthesia (p = 0.772, Figure 3c).

## Discussion

In the excision of large lipomas, concerns have been raised regarding the excessive use of local anesthetics, and general anesthesia has been selected in some cases [4]. To address this issue, tumescent anesthesia--using highly diluted lidocaine to allow for large injection volumes while minimizing systemic toxicity--has been reported as effective [4,6]. However, the relationship between lipoma size and the amount of local anesthetic used has not been sufficiently investigated. While the finding that larger or deeper lipomas require greater anesthetic volume is clinically intuitive, this study provides quantitative evidence supporting the safe use of highly diluted lidocaine even under such conditions. By demonstrating adequate analgesia despite reduced lidocaine concentration, our findings extend existing knowledge on the practical limits of tumescent local anesthesia in soft tissue surgery.

In this study, larger lipomas tended to be managed with higher dilution ratios of local anesthetics, and a positive correlation was observed between tumor size and the total anesthetic volume administered. In contrast, no association was found with the absolute amount of 1% lidocaine, and the efficiency per tumor volume was highest in the 10-fold dilution group. This suggests the potential to maintain analgesia while limiting the total dose of lidocaine. Tumescent anesthesia, originally developed for liposuction, has since been widely applied in dermatologic surgery [7,8]; however, reports on the efficacy of extreme dilution remain limited. The present study provides evidence to support this approach.

Moreover, it became clear that lipoma size was not the primary determinant of anesthetic choice; rather, tumor depth played a more critical role. In particular, deep-seated lipomas located between muscles or in proximity to major vessels and nerves tended to be managed under general anesthesia [5]. In this study, infiltration of 10-fold diluted lidocaine around the tumor proved effective, representing a safer and more versatile approach than intratumoral injection techniques reported in previous studies [1,6,9]. Taken together, these findings suggest that 10-fold diluted lidocaine may serve as a safe and practical anesthetic strategy for the excision of large lipomas.

This study has several limitations. The sample size was small and derived from a single institution, and tumor volume was estimated using an approximation. Therefore, the statistical power was limited, and the observed correlations should be interpreted with caution. Although all procedures were performed using a field block technique, the amount and pattern of initial anesthetic injection were not fully standardized, which may have affected the need for additional anesthesia and the interpretation of dose-response relationships. Operator-dependent factors, such as surgeon experience, use of magnification loupes, and dissection technique, were not controlled and may have influenced anesthetic volume or perceived surgical difficulty. Histologic or imaging characteristics, such as the presence of a fibrous capsule, were also not evaluated. Other structural or tissue-related factors may also influence the ease of excision and anesthetic requirements, suggesting that tumor depth alone may not be the best predictor. The number of deep-seated lipomas was limited, making conclusions regarding these cases less definitive. Moreover, general anesthesia remains appropriate in certain situations, and local anesthesia cannot always be considered optimal. Further studies are warranted to assess the efficacy of dilutions beyond 10-fold.

## Conclusions

Our findings suggest that 10-fold diluted 1% lidocaine provides sufficient anesthesia for excising large lipomas, thereby reducing the need for general anesthesia. Lipoma size correlated with anesthetic volume, but not with the absolute dose of lidocaine used. However, deeper lipomas were more likely to require general anesthesia due to surgical complexity. Using diluted lidocaine via a field block around the tumor offers a safe and effective strategy, especially when malignancy cannot be ruled out. Adoption of this method may expand the indications for local anesthesia in dermatologic surgery.
